# Mepolizumab treatment in a child with inherited TYK2 deficiency

**DOI:** 10.70962/jhi.20250106

**Published:** 2025-07-29

**Authors:** Aurélia Alimi, TYK2-consortium, Vivien Béziat, Jean-Laurent Casanova, Audrey Dupond-Athenor, Iris Fagniez, Ji Eun Han, Jean-Emmanuel Kahn, Boris Laccara, Gauthier Loron, Jerome Rambaud, Capucine Picard, Stéphanie Wanin, Stéphanie Boisson-Dupuis, Paul Bastard

**Affiliations:** 1 Department of Pediatric Hematology, Armand Trousseau University Hospital, Groupe Hospitalier Universitaire, AP-HP Sorbonne-University, Paris, France; 2 Department of Pediatric Allergology, Armand Trousseau University Hospital, Groupe Hospitalier Universitaire, AP-HP Sorbonne-University, Paris, France; 3 Laboratory of Human Genetics of Infectious Diseases, Necker Branch, INSERM U1163, Necker Hospital for Sick Children, Paris, France; 4 Paris Cité University, Imagine Institute, Paris, France; 5 St. Giles Laboratory of Human Genetics of Infectious Diseases, Rockefeller Branch, The Rockefeller University, New York, NY, USA; 6 Pediatric Hematology-Immunology and Rheumatology Unit, Necker Hospital for Sick Children, AP-HP, Paris, France

## Abstract

A child with inherited TYK2 deficiency and severe respiratory viral infections showed significant clinical improvement following treatment with mepolizumab. This report demonstrates that anti–IL-5 therapy can successfully manage Th2-driven inflammatory manifestations associated with complete TYK2 deficiency.

## Teaser

We report the case of a patient with complete TYK2 deficiency and virally induced hypereosinophilia and respiratory failure who responded to IL-5 blockade. TYK2 may be involved in regulating the Th1/Th2 balance in favor of Th1 and downregulating eosinophil recruitment in the airway.

**Figure 1.**
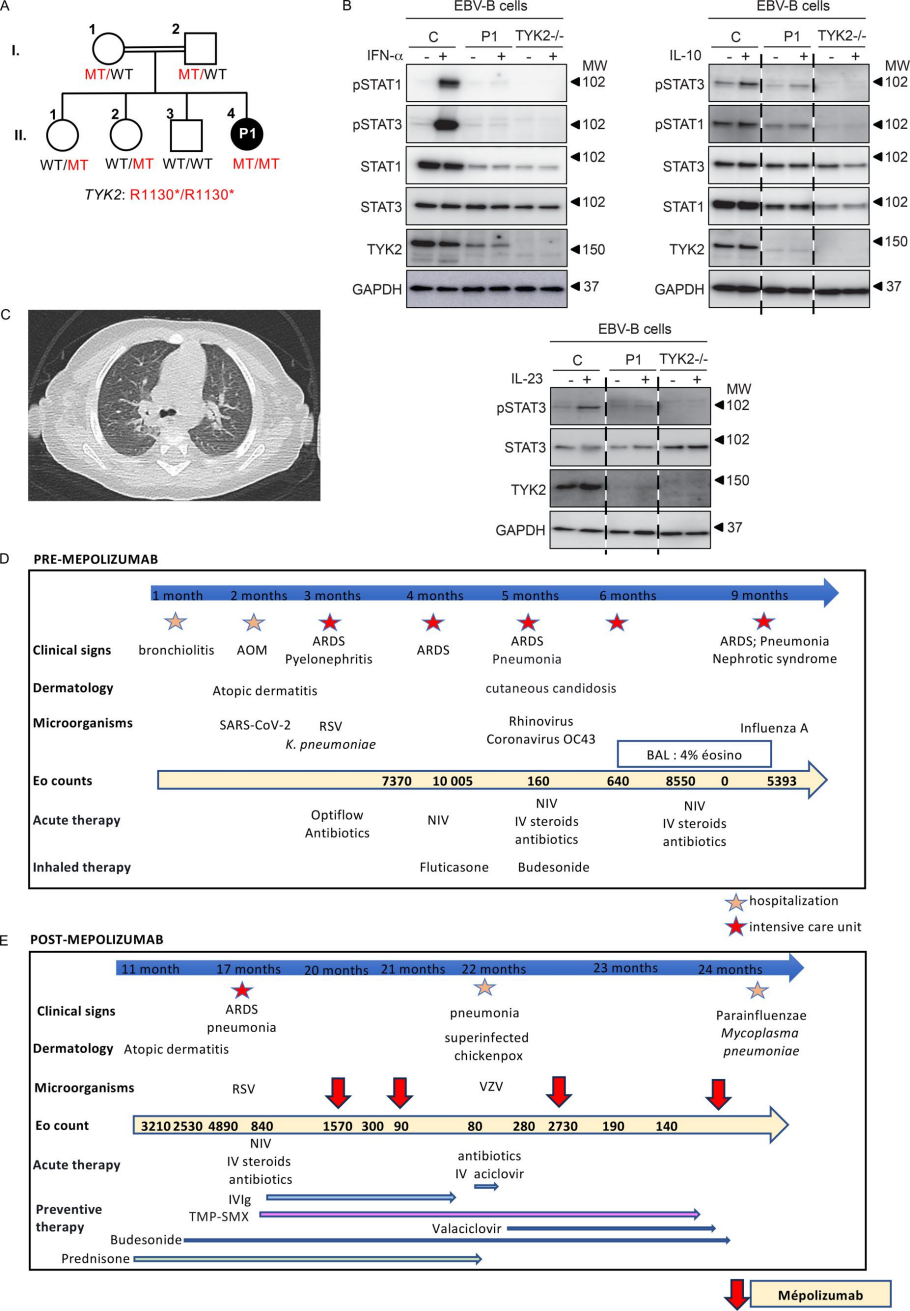
**Clinical and cellular characteristics of the TYK2 deficient patient. (A)** Family pedigree showing the segregation of the *TYK2* mutant (MT) allele. Double lines connect the two consanguineous parents. The closed black symbol indicates the proband (patient 1, P1) with TYK2 deficiency, and the open symbols indicate healthy family members. WT: wildtype. **(B)** Western blotting of the EBV-B cells from P1, showing TYK2 deficiency. The TYK2, STAT1, STAT3, pSTAT1, pSTAT3, and GAPDH proteins are shown. The response to IFN-α, IL-23 and IL-10 was similar to that in patients with complete TYK2 deficiency. MW, molecular weight in kD. **(C)** Computed tomography of P1 showing bilateral lung opacities. **(D)** Initial outcome before mepolizumab treatment. RSV: respiratory syncytial virus; BAL: bronchoalveolar lavage; NIV: noninvasive ventilation; IV: intravenous*.* AOM: acute otitis media. ARDS: acute respiratory distress syndrome*.* VZV: varicella zoster virus. **(E)** Outcome after mepolizumab treatment. IV IgG: intravenous immunoglobulin; TMP-SMX: trimethoprim-sulfamethoxazole*.* Source data are available for this figure: SourceData F1.

We report the case of a 2-year-old child born to consanguineous parents of Algerian descent who, from the age of 1 months old (mo), developed recurrent severe respiratory viral infections (Fig. 1 A). During the first 2 years of her life, she was hospitalized nine times including six in the intensive care unit for virus-induced acute respiratory distress, with pneumonia due to severe acute respiratory syndrome coronavirus 2 (SARS-CoV-2), respiratory syncytial virus, coronavirus OC43, and influenza A virus (IAV). Each of these episodes was followed by hypereosinophilia (Fig. 1 D). The patient soon developed recurrent episodes of severe acute respiratory failure with recurrent episodes of wheezing, without other clinical signs of atopy (Fig. 1 D). During each hospitalization for acute respiratory failure, she received systemic steroid treatment, leading to a decrease in eosinophil counts and respiratory improvement. From the age of 4 mo onward, the patient had hypereosinophilia (up to 10,000/mm^3^) but with normal total IgE levels (Fig. 1 D). Bilateral lung opacities were observed (Fig. 1 C), and bronchoalveolar lavage at the age of 8 mo revealed no pathogens and the presence of 4% eosinophils (while under steroid treatment). Treatment with corticosteroids at a dose of at least 1 mg/kg/day was required to obtain an eosinophil count of 2,500/mm^3^ after a 12-mo period of recurrent hospitalizations, but the patient nevertheless continued to suffer from episodes of virus-induced respiratory distress. The patient had no atopic or parasitic disease that could explain the hypereosinophilia (toxocariasis serology and stool parasitology tests were negative), and hematological causes of hypereosinophilia were ruled out by an otherwise normal hemogram and a bone marrow aspirate that was normal (including karyotype and reverse transcriptase multiplex ligation-dependent probe amplification (RT-MLPA) transcripts: negative for Janus kinase 2, FIP1, and breakpoint cluster region Abelson murine leukemia [BCR ABL]) except for 14% hypereosinophilia. Blood immunophenotyping showed: 1.3% CD3^+^CD4^+^CD7^−^ (<physiological threshold of 3%), 0.4% CD3^−^CD4^+^CD7^−^CD2^+^CD5^+^ (<physiological threshold of 1%), and 1.5% CD3^+^CD4^−^CD8^−^TCR α and β (<physiological threshold of 1.5%) cells. There was, therefore, no phenotypic evidence for a lymphoid origin of the eosinophilia. Immunophenotyping showed slightly low percentages of naïve CD4 and CD8 T cells and central memory CD8 T cells, a high percentage of memory effector CD8 T cells and a normal percentage of terminally differentiated effector memories, mild B lymphocytosis with a high percentage of CD19^+^/CD27^+^ B cells, and natural killer (NK) lymphocytosis. Compared with aged-matched controls, deep immunophenotyping by cytometry by time-of-flight also showed a reduced counts of naïve CD4 (679/µl versus a mean of 1,283/µl) and CD8 T cells (354/µl versus a mean of 607/µl) and an increased count of Th2 cells (CCR4+CCR6^−^ CD4 T cells: 120/µl versus a mean of 43/µl). Functional assays showed normal T cell proliferation in response to phytohemagglutinin and OKT3 (normal value > 30%), but no T cell proliferation in response to tetanus toxoid and candidin. Interleukin 5 (IL-5) was undetectable in blood. Postvaccination serological test results were normal for diphtheria, tetanus, pneumococcus, and *Haemophilus influenzae*. Complement levels were normal. A tryptase test was negative. IgG, IgA, and IgM levels were within the normal ranges. IgE levels were 21 kIU/L (normal). We then performed panel sequencing for eosinophilia, which revealed a biallelic variant of *TYK2* (encoding tyrosine kinase 2) that was also identified by whole-exome sequencing (WES), (c.3388C>T, p.Arg1130*). No other candidate variant was identified by WES. This variant was predicted to be loss-of function due to the creation of a premature stop codon in the C-terminal part of TYK2, and it was not reported in gnomAD V4.1.

P1’s variant was confirmed by Sanger sequencing. Both her parents and her two sisters were heterozygous for the variant, and her brother was WT and healthy. We investigated the functional impact of the variant, using EBV-B cells derived from P1. Residual amounts of TYK2 protein were detected on western blots, but at a slightly lower molecular weight. Nevertheless, responses to IL-23, interferon (IFN)-α, and IL-10 were as weak as those in a patient with complete TYK2 deficiency (Fig. 1 B), suggesting that the patient displayed autosomal recessive (AR) complete TYK2 deficiency, with residual protein expression, as previously described (1). TYK2 is one of the four human JAKs. It is involved in the IL-10, IL-12, IL-23, and type I IFNs (13 IFN-α subtypes, IFN-ω, IFN-β, IFN-ε, and IFN-κ) pathways. Complete TYK2 deficiency was first described in 2006 in a single patient, and five forms of AR TYK2 deficiency have now been described in 25 patients: (1) complete without and (2) with residual expression, (3) partial deficiency affecting all pathways, partial deficiency affecting specifically IL-23 signaling due to (4) rare and (5) common variants. In these patients, impaired IL-12– and IL-23–mediated IFN-γ production underlie mycobacterial diseases due to tuberculous and nontuberculous mycobacteria. Like patients with IL-12Rβ1 deficiency, in whom IL-12– and IL-23–mediated IFN-γ production is abolished, some TYK2-deficient patients are also susceptible to intramacrophagic pathogens (*Salmonella*). Their IL-23–dependent induction of IL-17 is also weak, accounting for their fungal diseases (*Candida*). Impaired responses to type I IFNs underlie severe viral diseases, including COVID-19 pneumonia, influenza pneumonia, herpes simplex encephalitis, and adverse reactions to live attenuated vaccines. Impaired responses to IL-10 seem to be clinically silent. Incomplete clinical penetrance has been observed for mycobacterial and viral diseases, as 48% and 60% of patients, respectively, develop these diseases. Deep immunophenotyping revealed no peripheral blood mononuclear cells (PBMC) abnormalities in patients with the various forms of TYK2 deficiency, indicating the presence of normal numbers and percentages of the different myeloid and lymphoid cell subsets (purely adaptive T cells [CD4^+^ T, CD8^+^ T cells, and their subsets], innate-like adaptive T cells [γδ T, mucosal-associated invariant T, and invariant NK T cells], and innate lymphoid cells [NK, innate lymphoid cell progenitors, and ILC2]), monocytes, and dendritic cells in three TYK2-deficient patients (1) and in our patient.

Eosinophil levels were rarely mentioned (2). Our patient presented virus-triggered lung hyperreactivity and severe hypereosinophilia with very deleterious effects on her quality of life. She did not suffer from the mycobacterial, fungal, or bacterial infections described in previously reported patients (1), but her susceptibility to a broad range of viral diseases was explained by defective type I IFN responsiveness. This defect was probably a triggering factor in her secondary lung hyperreactivity, which became her main condition, perhaps driven by defective Th1 and excessive Th2 differentiation, as previously suggested (3). Given the patient’s history of severe viral diseases, preventive management with infusions of polyvalent immunoglobulin (IVIg) and trimethoprim-sulfamethoxazole in addition to inhaled corticosteroids was initiated (Fig. 1 E). IL-5 and IL-6 plasma levels were normal. Other cytokine levels were not measured. However, due to the dependence on systemic corticosteroids and the recurrent pulmonary symptoms and hypereosinophilia, we decided to initiate targeted corticosteroid-sparing therapy to block IL-5 (Fig. 1 E) (4). Mepolizumab, used for the treatment of severe eosinophilic asthma, eosinophilic granulomatosis, and hypereosinophilic syndrome, was started at the age of 20 mo, at a dose of 40 mg per month delivered subcutaneously. This treatment was well tolerated clinically. Two weeks after the first injection, eosinophil counts had fallen strongly, to 300/mm^3^, reaching normal levels one month later. The patient suffered from chickenpox due to varicella zoster virus infection, leading to a suspension of oral steroid treatment, and had one episode of respiratory distress due to infection with parainfluenza virus and *Mycoplasma pneumoniae* but without hypereosinophilia after treatment initiation. This patient with complete TYK2 deficiency, who suffered from severe viral infections and severe hypereosinophilia causing wheezing respiratory disease, is now three years old and remains clinically well on mepolizumab and IVIg treatment, with an eosinophil count of 140/mm^3^ (Fig. 1 E).

Thus, we report the case of a patient with complete TYK2 deficiency and virally induced hypereosinophilia and respiratory failure who responded to IL-5 blockade. Routine assessment of circulating levels of IL-5 and/or deep immunophenotyping showing a clear skewing toward a Th2 phenotype can guide therapeutic intervention toward the use of targeted therapies, such as treatments targeting IL-5. The mechanism underlying this abnormal skew toward a Th2 phenotype has yet to be fully elucidated, but studies in mice suggest that Tyk2 may be involved in regulating the Th1/Th2 balance in favor of Th1 and downregulating eosinophil recruitment in the airway (5). A similar mechanism may be at work in our patient.

## Supplementary Material

Table S1 lists the TYK2-consortium members and their affiliations.

SourceData F1 is the source file for Fig. 1.
