# Supplementary material for: Mepolizumab treatment in a child with inherited TYK2 deficiency
Source: J Hum Immun. 2025 Jul 29;1(3):e20250106. doi: 10.70962/jhi.20250106 (PMC12425477; doi:10.70962/jhi.20250106)
Supplement: Table S1 — lists the TYK2-consortium members and their affiliations. [file jhi_20250106_tables1.pdf]

TYK2-consortium*

Vivien Béziat[1-4], Jean-Laurent Casanova[1-5], Audrey Dupond-Athenor[6], Iris Fagniez[1], Ji Eun Han[1], Jean-Emmanuel Kahn[7], Boris Laccara[8], Gauthier Loron[9], Jerome Rambaud[10], Capucine Picard[3,5,11]

[1]Howards Hugues Medical Institute, New-York, USA

[2]Laboratory of Human Genetics of Infectious Diseases, Necker Branch, INSERM U1163, Necker Hospital for Sick

Children, Paris, France.

[3]Paris Cité University, Imagine Institute, Paris, France.

[4]St. Giles Laboratory of Human Genetics of Infectious Diseases, Rockefeller Branch, The Rockefeller University,

New York, NY, USA.

[5]Pediatric Hematology-Immunology and Rheumatology Unit, Necker Hospital for Sick Children, Assistance Publique-Hôpitaux de Paris (AP-HP), Paris, France, EU.

[6]Department of Pediatric Pulmonology, Armand Trousseau University Hospital, Groupe Hospitalier Universitaire, AP-HP Sorbonne-University, Paris, France

[7]Department of Internal Medicine, Hôpital Ambroise Paré Boulogne Billancourt, Université Versailles Saint Quentin en Yvelines, Versailles, France.

[8]Department of Intensive Care, Robert Debre University Hospital, Groupe Hospitalier Universitaire, AP-HP Sorbonne-University, Paris, France

[9]Department of Intensive Care, Reims University Hospital, Groupe Hospitalier Universitaire, Champagne, France

[10]Department of Intensive Care, Armand Trousseau University Hospital, Groupe Hospitalier Universitaire, AP-HP Sorbonne-University, Paris, France

[11]Study Center for Primary Immunodeficiencies, Necker Hospital for Sick Children, Assistance Publique-Hôpitaux de Paris (AP-HP), Paris, France, EU.
