# Supplementary figures and images for: Mepolizumab treatment in a child with inherited TYK2 deficiency
Source: J Hum Immun. 2025 Jul 29;1(3):e20250106. doi: 10.70962/jhi.20250106 (PMC12425477; doi:10.70962/jhi.20250106)

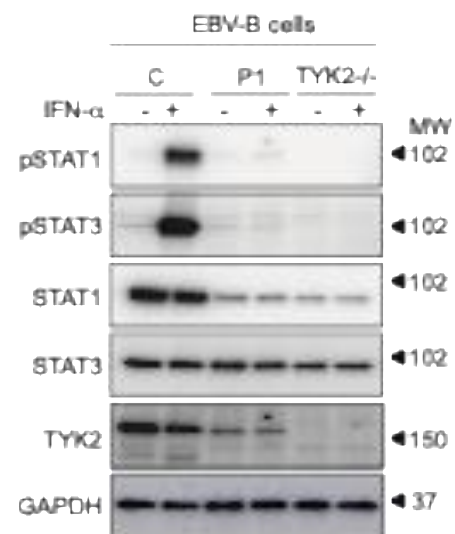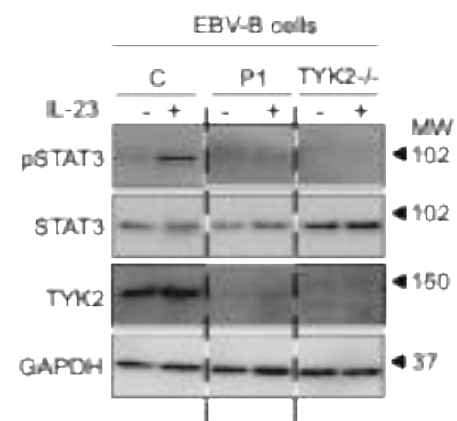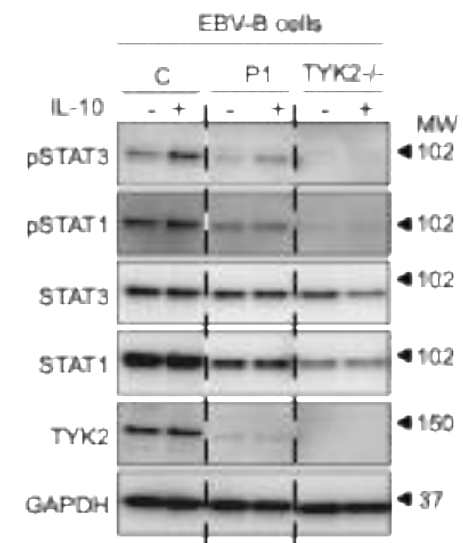

Figure 1  
Panel B  
IFNa stimulation

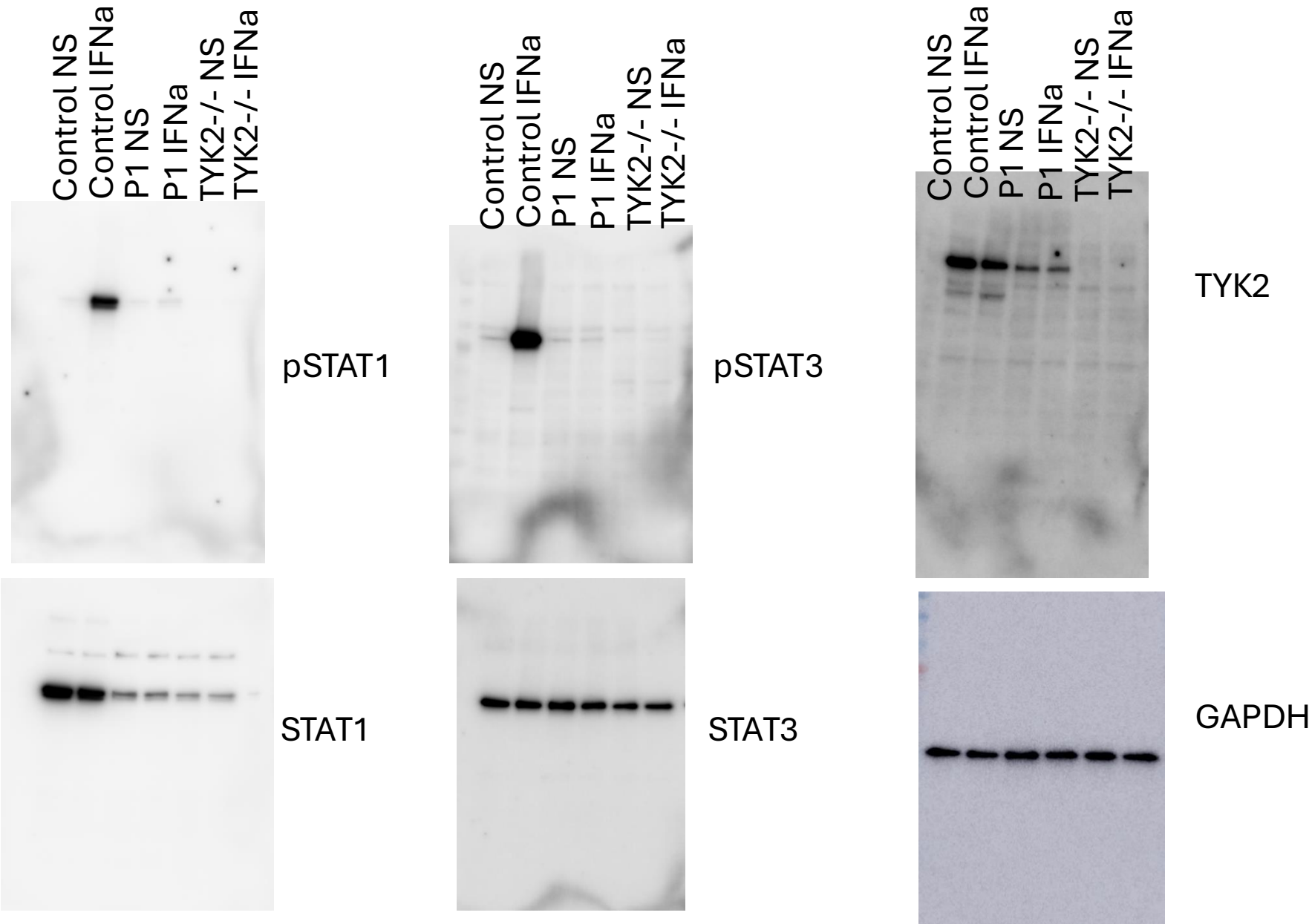

Figure 1  
Panel B  
IL-23 stimulation

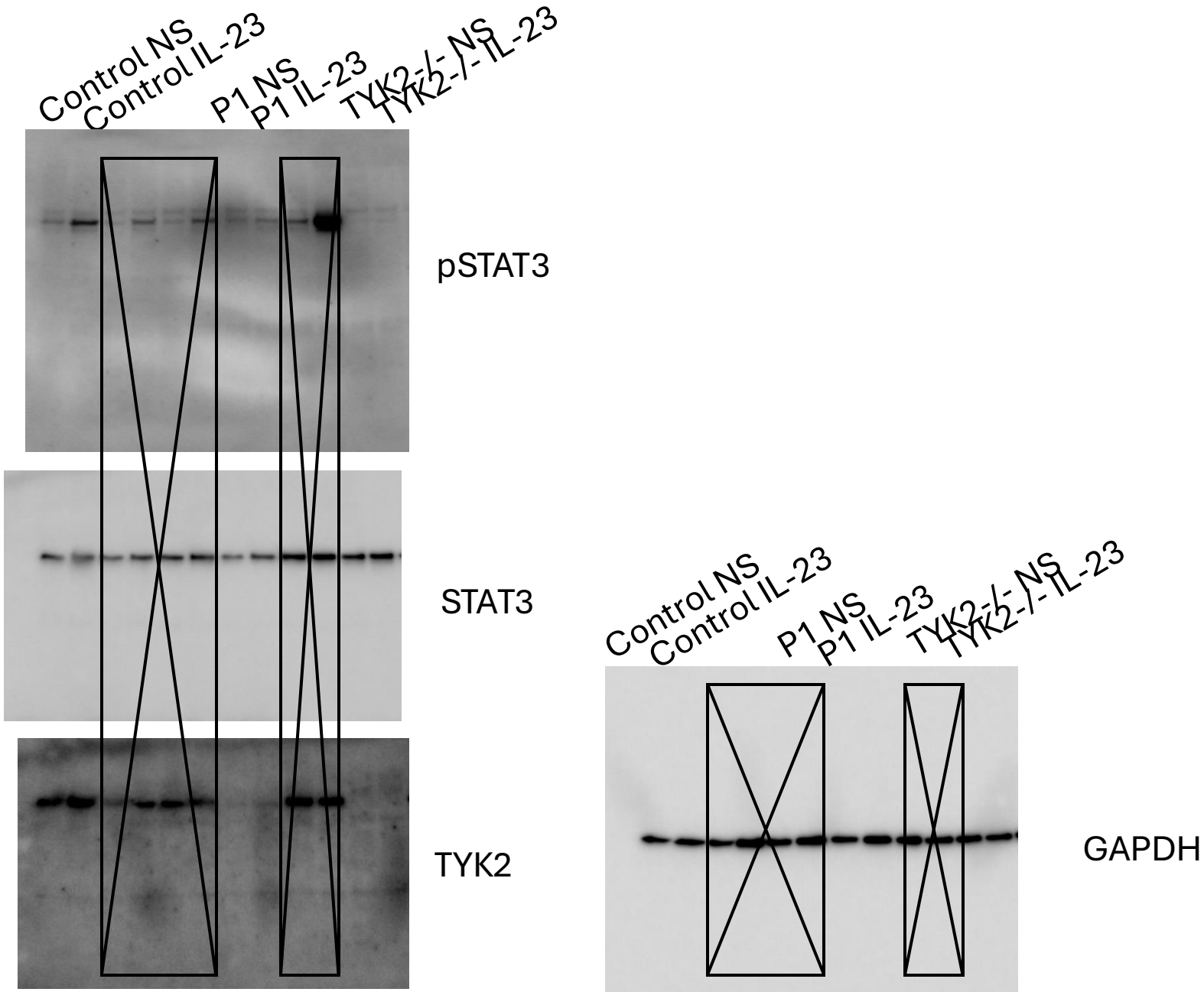

Figure 1  
Panel B  
IL-10 stimulation

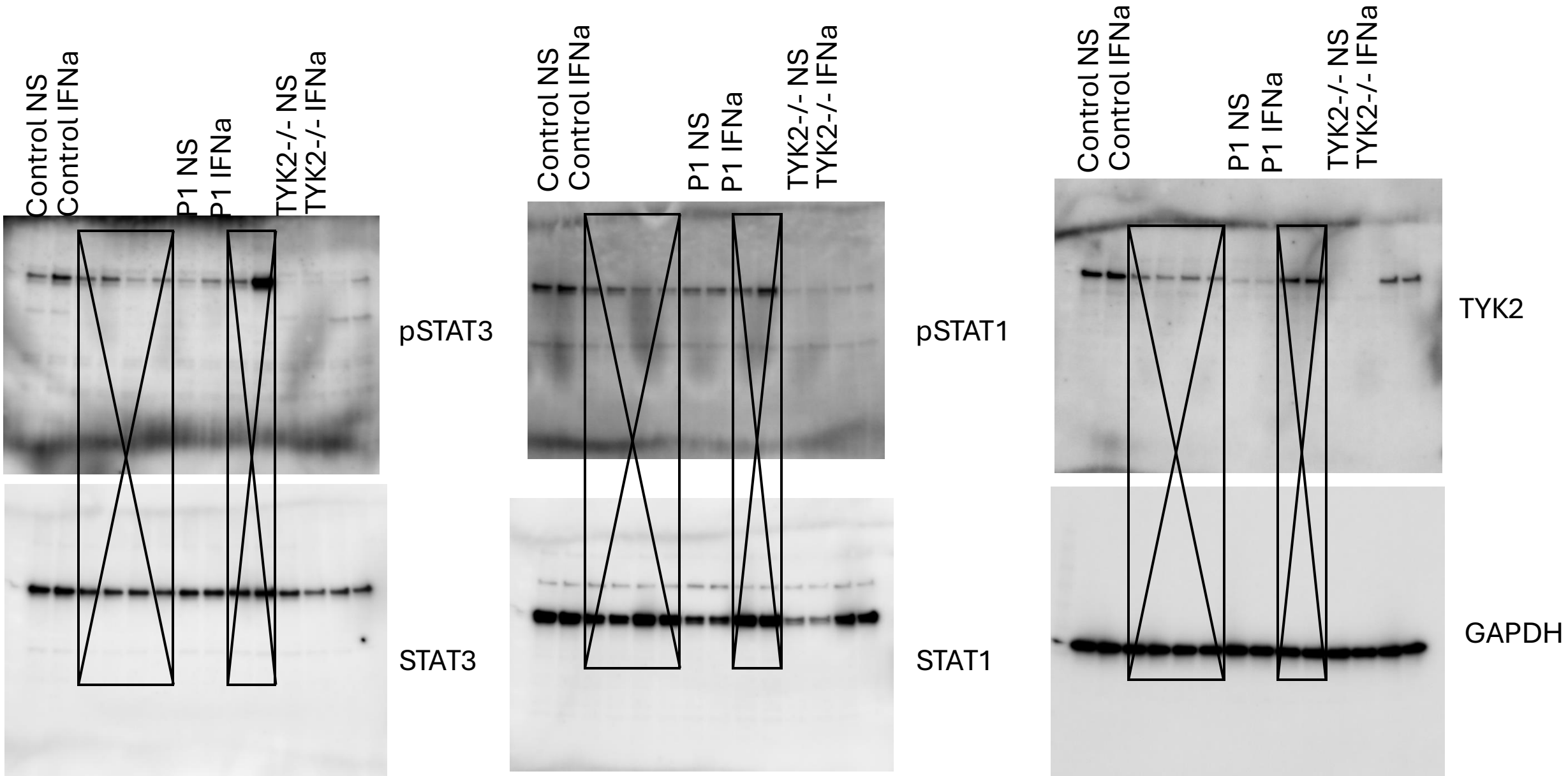

Supplement: SourceData F1 — is the source file for Fig. 1. [file jhi_20250106_sourcedataf1.pdf]
